# Analysis of accessibility to emergency rooms by dynamic population from mobile phone data: Geography of social inequity in South Korea

**DOI:** 10.1371/journal.pone.0231079

**Published:** 2020-04-08

**Authors:** Sung Bum Yun, Soohyun Kim, Sungha Ju, Juhwan Noh, Changsoo Kim, Man Sing Wong, Joon Heo

**Affiliations:** 1 Department of Civil and Environmental Engineering, Yonsei University, Seoul, South Korea; 2 Department of Preventive Medicine, Yonsei University, Seoul, South Korea; 3 Department of Land Surveying and Geo-Informatics, Hong Kong Polytechnic University, Kowloon, Hong Kong; 4 Research Institute for Sustainable Urban Development, The Hong Kong Polytechnic University, Hung Hom, Hong Kong; University of Wisconsin Madison, UNITED STATES

## Abstract

Accessibility of emergency medical care is one of the crucial factors in evaluating national primary medical care systems. While many studies have focused on this issue, there was a fundamental limit to the measurement of accessibility of emergency rooms, because the commonly used census-based population data are difficult to provide realistic information in terms of time and space. In this study, we evaluated the geographical accessibility of emergency rooms in South Korea by using dynamic population counts from mobile phone data. Such population counts were more accurate and up-to-date because they are obtained by aggregating the number of mobile phone users in a 50-by-50 m grid of a locational field, weighted by stay time. Considering both supply and demand of emergency rooms, the 2-step floating catchment analysis was implemented. As a result, urban areas, including the capital city Seoul, showed lower accessibility to emergency rooms, whereas rural areas recorded higher accessibility. This result was contrary to the results analyzed by us based on census-based population data: higher accessibility in urban areas and lower in rural. This implies that using solely census data for accessibility analysis could lead to certain errors, and adopting mobile-based population data would represent the real-world situations for solving problems of social inequity in primary medical care.

## Introduction

Inaccessibility of health facilities could be a reflection of social inequality or injustice in public health [1]. For example, some individuals in South Korea cannot have their medical care needs met, due to the lack of geographical accessibility of medical services, although most public healthcare services have been held accountable by the Korean government for a long time [2]. Recent studies found that the probability of mortality after an emergency was affected by the distance from an emergency hospital [3, 4]. Social inequity in public health has been a challenging issue in social and community welfare, and many researchers have examined the geographical aspects of such an issue [5, 6]. In the field of emergency medical services (EMS), inequity was considered a factor for deciding EMS location or evaluating the entire EMS system [7, 8].

‘Accessibility analysis’ has been frequently used to identify geographical balance for various public facilities. Geographical status was simply described on maps by pinpointing some areas with low accessibility or visualizing community facilities, such as hospitals, green spaces, and public transit [9–11]. Also, areas of restricted access to distribution centers were identified [12], new locations for public transit stations were selected [13], and accessibility of green spaces in suburban areas was analyzed as advanced analyses [14, 15].

Various GIS-based methods have been implemented for the purpose of accessibility analysis [16]. Agent-based simulations of human movements in natural disaster management have been used to provide locations for temporary emergency services [17]. Fuzzy C-means Clustering and particle swarm optimization-based methods have been utilized to derive optimal locations for common service centers [18]. Bayesian belief networks and hybrid genetic algorithms have also been used to derive optimal locations for delivery-service distribution centers [19, 20]. However, these methods were not suitable for consideration of both supply and demand for such facilities and did not fully describe real-world situations.

To overcome such simplification, 2-Step Floating Catchment Analysis (2SFCA) algorithms [21] were utilized. For example, accessibility maps were derived for medical facilities to determine where new medical facilities should be located [22]. The spatial accessibility of primary care services in rural areas were measured based on the various sizes of a circle-shaped buffer [23] by using Australian official population data as a demand of the facility, and a similar study was conducted for fatal motor vehicle crashes in Texas, USA [24], where Euclidean distance was used for accessibility measurements. Those two studies executed the implementations at various time intervals. However, the circular buffer or Euclidean distance in those studies was simplistic and formulaic to an extent and, for this reason, transportation networks are more often used for delineating service areas for accessibility analysis. A recent study in China used network data to find network-based service areas and to determine the accessibility of medical care in Sichuan, China [25]. The areas with low accessibility to police stations were detected, and optimal locations for new facilities were decided.

Even when supply and demand sides were considered with real road network data, there was still a fundamental limitation of measuring accessibility, which was caused by the source of data. In other words, if census-based population data were used, the population count from the census hardly reflected the demand of the facility. Although censuses are by far the most important source of demographic data, such data are occasionally collected, and it is difficult to reflect dynamic patterns in time and space in detail [26]. Some population estimation methods were developed to produce more accurate and contemporary data from census information, but, even in population estimation, more fine-scale spatial units for census input data were better in reducing estimation errors [27, 28].

Mobile-based population data presented in this study can be an alternative to a real population count for accessibility analysis, which is now available due to technological advances in wireless communication networks and a high coverage of mobile phone subscriptions [29, 30]. Originally, mobile phone data were operational data collected by cell towers that contained the location information of every mobile phone. Such data could then be transformed to a more specific type of data, including population counts [31]. Population density estimation based on mobile phone data has been developed [29, 30, 32–34], and practical implementation of this type of population data has been attracting attention [29–31]. This population count meets the concept of ‘service population count’, which is the count that includes “some or all of the difficult-to-enumerate groups, depending on the type of service population required,” according to the United Nation's recommendations for population census [35]. However, the application of such data has been rarely studied, due to the difficulties of data acquisition, and there is little research in accessibility analysis [36].

In the present study, a geographical accessibility analysis for emergency medical care facilities were conducted based on mobile-based population counts collected using the 2-Step Floating Catchment Analysis (2SFCA) algorithms. We conjectured that the population data from mobile phone would more accurately describe real situations, rather than the population data from census. Mobile-based data recognized people's current location and reflected the everyday reality of people whose location changes according to activity, purpose, and time use allocation, unlike the census that collected usual residence. As traffic develops, individuals are more likely to be in different locations during the day and at night, or on weekdays and weekends, and this new form of data can better explain the change in location.

Using population data from mobile network, this study tests the following hypotheses. First, is there a difference of accessibility in emergency rooms in South Korea between using census-based data and mobile-based data for demand of emergency room? Specifically, is there a difference in the geographical distributions of accessibility between the two cases? Second, is there any difference in accessibility between setting the driving time of the vehicle to 10 minutes and 15 minutes to derive service areas as a supply of emergency rooms? Our results from two types of population data will be compared to explain a better solution for policy making with respect to the spatial distribution of emergency medical care centers.

## Methods

**Fig 1:**
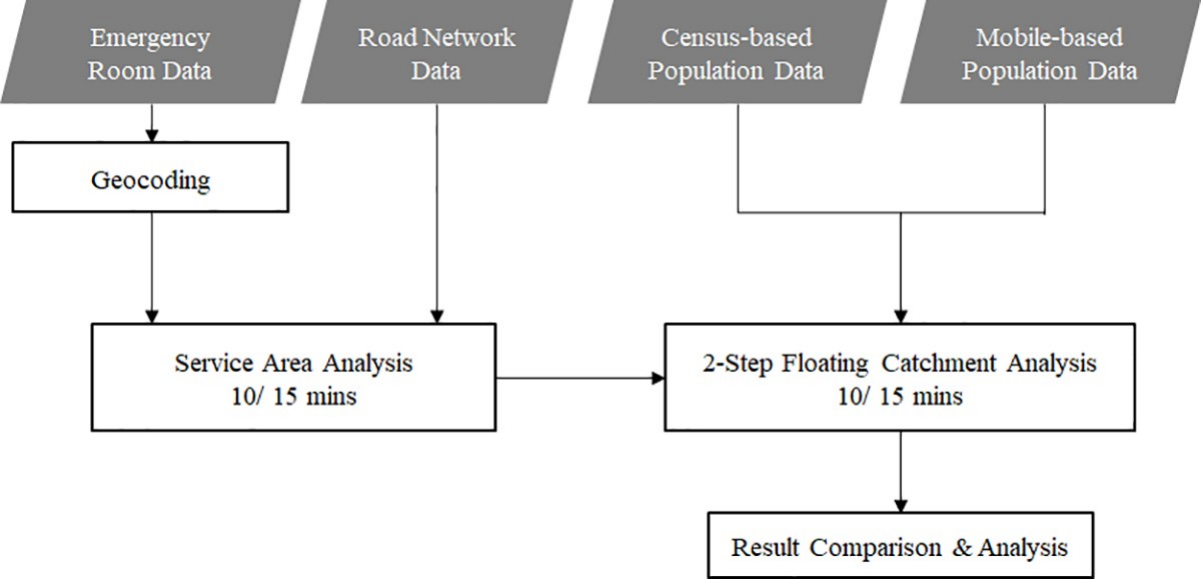
Flowchart of the study.

Four types of datasets, such as emergency room data, road network data, mobile-based population data, and census-based population data, were used in this study. A service area analysis was performed using road network data in order to derive the service area of ‘to’ and ‘from’ the facilities. Relying on such service areas, a 2SFCA analysis was conducted twice using both census and mobile-based population data, in order to draw the accessibility score map. The flow of the study was summarized in Fig 1.

### Study area

**Fig 2:**
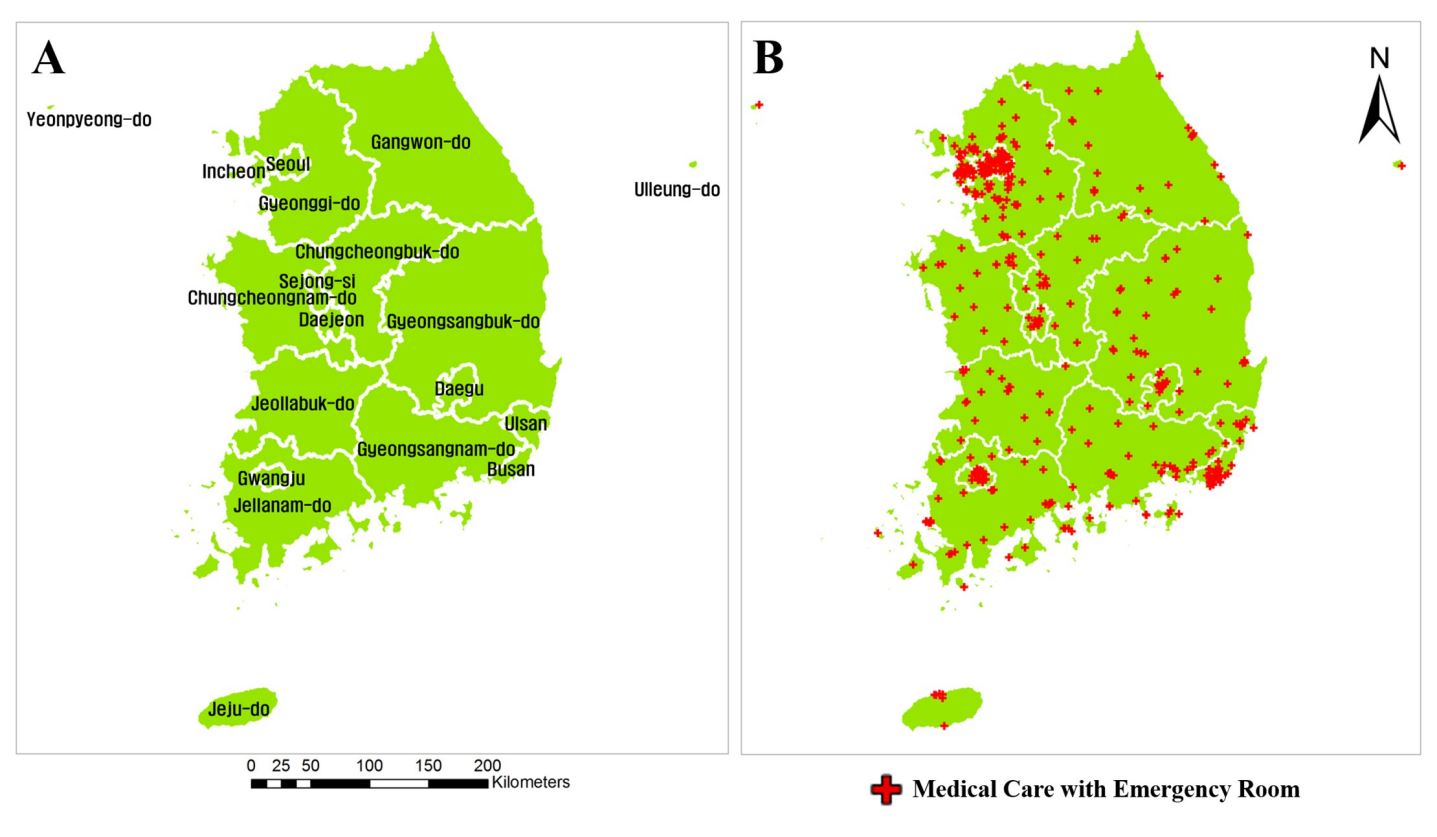
Map of South Korea. (A) Administrative district. (B) Locations of emergency rooms.

South Korea is a major research area of 51 million population with emergency rooms in 424 hospitals. South Korea’s capital Seoul and the Seoul metropolitan area were considered to be separate study areas due to their large populations. Fig 2A shows the names of the provinces & administrative districts applicable to this study.

### Datasets

The emergency room data were obtained from the E-Gen website, serviced by the Ministry of Health and Welfare [37]. The data contain the names and addresses of hospitals with emergency rooms and the available capacity for patients (Table 1). Fig 2B indicates, with red crosses, the spatial locations of the hospitals with emergency rooms. Additionally, the road network data were obtained from the Intelligent Transport System (ITS) website, serviced by the Ministry of Land, Infrastructure and Transport, as was the map of South Korea [38].

**Table 1:** Data dictionary for medical care with emergency room.

Data Category	Column Name	Description	Example or category
Emergency room data	HP_NM	Name of the Hospital with Emergency Room	Severance Hospital, Seoul
	ADD	Address description	Seoul, Seodaemun-Gu, Yonsei-ro, 50–1
	TOT_CAP	Capacity of Emergency Room	42
	CU_CAP	Current Available ER Capacity	0
	ER_QUAR	ER capacity for Quarantine	1
Census-based population data	CD	Administrative district code ‘Dong’	000000024
	AGE	Age Group	0~9/ 10~19/ 20~29/ 30~39/ 40~49/ 50~59/ 60~69/ Over 70
	TIME	Survey time	201604
Mobile-based population data	CD	Grid Code	000000024
	XCD	X Coordinate in UTM-K	950597.8311
	YCD	Y Coordinate in UTM-K	1951576.3362
	GEN	Gender	0 : Male / 1 : Female
	AGE	Age Group	0~4/ 5~9/ 10~14/ 15~19/ … /60~64 /Over 65
	AVFP	Average Real Population per Month in 50m x 50m Grid	13.24

Census-based population data of 2015 were collected from the Korean Statistical Information Service (KOSIS) [39] (Table 1). The data contain the population of the administrative district, known as ‘Dong’, which is the 2^nd^ smallest administrative unit available after census block. Most large cities, such as Seoul and Busan, are considered as ‘Si’ administrative districts, which consist of multiple ‘Dongs’. Also, the census-based population of each 10-year age group was provided.

**Fig 3:**
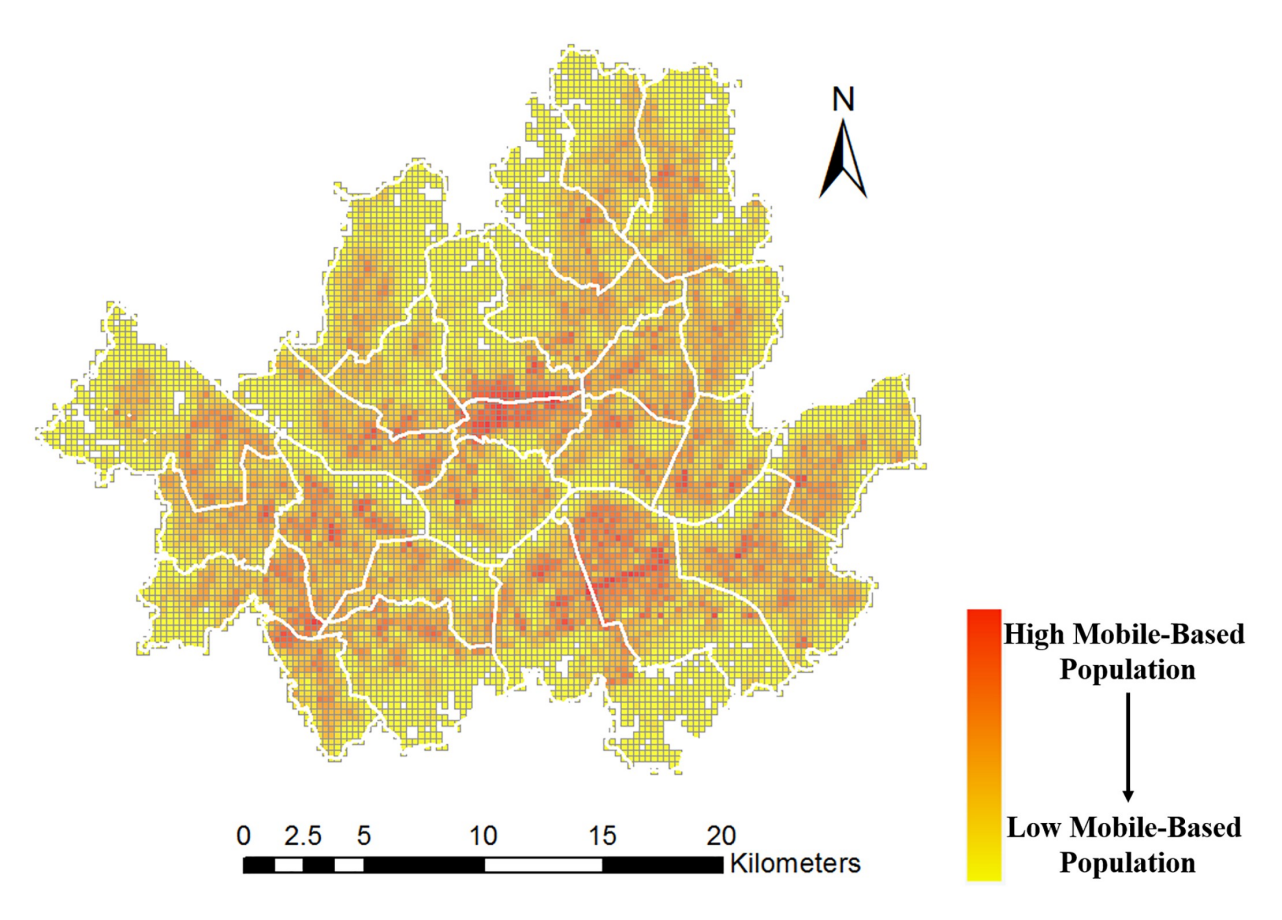
Average real population count of May 2015 in Seoul, South Korea.

The mobile-based population data were collected by the Korean mobile network, SK Telecom [40]. The data contain a per-month average population count within a 50m-by-50m grid covering all regions of South Korea, as well as age and gender of the user (Table 1). The population counts in each grid were measured every hour by using the location data of each cell phone, recognized by a cell tower in 1x/2G/3G/4G networks. In particular, the per-month average population count was obtained by counting how many and how long persons are situated in a given 50m x 50m grid. For example, if a person stays within the grid for a whole day (24 hours), he or she is counted as 1/30, i.e. 0.033, since the data themselves are monthly averaged. Fig 3 shows the average real population of the Seoul area in May 2015. The averaged population counts include both daytime and nighttime populations, reflecting changes over time in detail. Also, the counts include workers, tourists, business travelers, and some school children, who are considered difficult to enumerate.

The data processing company, SK Geovision, estimated the total number of real people counts using SK Telecom's data. SK Telecom's users make up 44.4% of total mobile phone users in South Korea, followed by other providers such as KT (28.2%) and Uplus (23.1%). In estimation, the correction factor per 'Dong' was obtained from the census-based population, and the factor then adjusted the known number of service subscribers in data to the total number of all who were at a particular place and time. The population data produced by SK Geovision have been widely used in various statistical implementations, such as location-based service ‘T-map.’ Also, they have provided information to local governments seeking better public services. The data were accepted as ‘official’ statistics data [39] and presented to the UN Global Compact as showcasing new possibilities for big data [40].

The census-based population data estimate the total population of Seoul as 51 million, whereas the mobile-based population estimates it as 46 million. The difference could be due to people who do not own a mobile phone, the usage rate of mobile phones in South Korea being around 95% [41]. If this 5% of the population without mobile phones is taken into account, half of the 5 million differences between the two results could be explained. The other 2.5 million could have been due to error in the estimation process related to people who use other telecommunication services, such as ‘KT’ or ‘Uplus’. In terms of the calculation of accessibility indices for emergency rooms, the authors presumed the mobile-based population data along with the census-based population data to be sufficient.

We complied with the terms of service for the websites from which we collected data. Data were anonymized before access by the authors and there are no potential risks to individuals or individual privacy.

### Preprocessing

As noted above, because the emergency room data collected by the E-Gen contained only addresses, a geocoding process was required to convert addresses into the XY coordinates. The open-source geocoding tool provided by Biz-gis [42] was used along with Google Maps API in the procedure. The geocoding tool matched addresses in a text file with Google Maps’ XY coordinates generating an output of a shapefile or a text file.

### Network-based service area analysis

‘Network-Based Service Area Analysis’ is a method of service area delineation based on street networks. Unlike the traditional method of buffering a point of service center, which creates a circle, the network-based service area is an irregularly-shaped region that encompasses all the accessible streets within a given cost, such as time and distance. On-demand, urgent medical care is the primary purpose of emergency medical services (EMS). According to the ‘platinum’ and ‘golden’ time intervals that have been defined for EMS [43, 44], 5 to 10 minutes is regarded as ‘platinum time’ or ‘platinum 10 minutes’ within which emergency patients should reach medical care in order to minimize the chances of worsening injuries or mortality [43]. The time intervals of 10 and 15 minutes were used for network-based service area analysis in this study. To calculate the required time for travel, previous studies conducted on Korean emergency vehicle average speeds were reviewed. The average speed of an emergency vehicle in an urban area was 35 to 40 km/h, and for a rural area, 40 to 45 km/h [45]. For the present calculations, the authors used 40km/h as the emergency vehicle speed.

To implement 2SFCA, which requires a service area from both the supply side and the demand side, network-based service area analysis was conducted twice: first for ‘supply’, originating from each emergency care location, and second, for ‘demand’, originating from the centroid of each administrative district ‘Dong’.

### Accessibility analysis

Accessibility analysis allows for derivation of an accessibility index score for each administrative district. Recent studies frequently utilized 2SFCA and gravity model analysis [44, 45]. Since the present study’s main purpose was to compare accessibility results from census and mobile-based population data, a single method was selected. Gravity model analysis has a high dependency on *β* (distance decay) [46], and as such, when the distance decay factor is modified, the derived results differ markedly. Due to the difficulty of choosing the best distance decay factor representing the real world most correctly in a gravity model, 2SFCA analysis was utilized to provide the accessibility results [47]. 2SFCA is an algorithm for derivation of an accessibility index score by utilizing the supply and demand ratio to calculate, from given points, an accessibility index for various facilities [22]:

$$R_{j}=\frac{S_{j}}{\sum_{k\in \left\{d_{\mathrm{kj}}\leq d_{0}\right\}}D_{k}}$$ (1)

$$A_{i}^{F}=\sum_{j\in \left\{d_{\mathrm{ij}}\leq d_{0}\right\}}R_{j}=\sum_{j\in \left\{d_{\mathrm{ij}}\leq d_{0}\right\}}\left(\frac{S_{j}}{\sum_{k\in \left\{d_{\mathrm{kj}}\leq d_{\theta }\right\}}D_{k}}\right)$$ (2),

where *i* and *k* depict the unit demand area ‘Dong’, *k* being used for the ‘first-step’ and *i* for the ‘second-step’ of 2SFCA, and *j* is a supply center (i.e., emergency room) location. *d*_*0*_ is the threshold distance which implies a given threshold for network-based service areas, *d*_*kj*_ is the distance between demand area *k* and supply center *j*, and *d*_*ij*_ is the distance between demand area and supply center *j*. *D*_*k*_ is the demand for emergency rooms for this study’s census-based or mobile-based population within the service area, *S*_*j*_ is the supply potential (i.e., capacity) for emergency rooms, and *R*_*j*_ is the ratio of supply to demand for a specific supply facility [48]. In Eq (2), the *R*_*j*_ of all supply facilities is determined once more in order to provide *A*_*i*_^*F*^, which is the accessibility index score for each ‘Dong’ administrative district. Eq (1) derives the acceptability ratio of each hospital with emergency rooms by calculating supply over demand, which means the supply of emergency patient capacity with potential patients. Next, Eq (2) calculates accessibility of each hospital with emergency rooms for each ‘Dong’ district using the acceptability ratio derived in Eq (1). The catchment analysis threshold *d*_*0*_ was 10 minutes or 15 minutes as mentioned in network-based service area analysis.

## Results

### Comparison between census- and mobile-based populations

**Fig 4:**
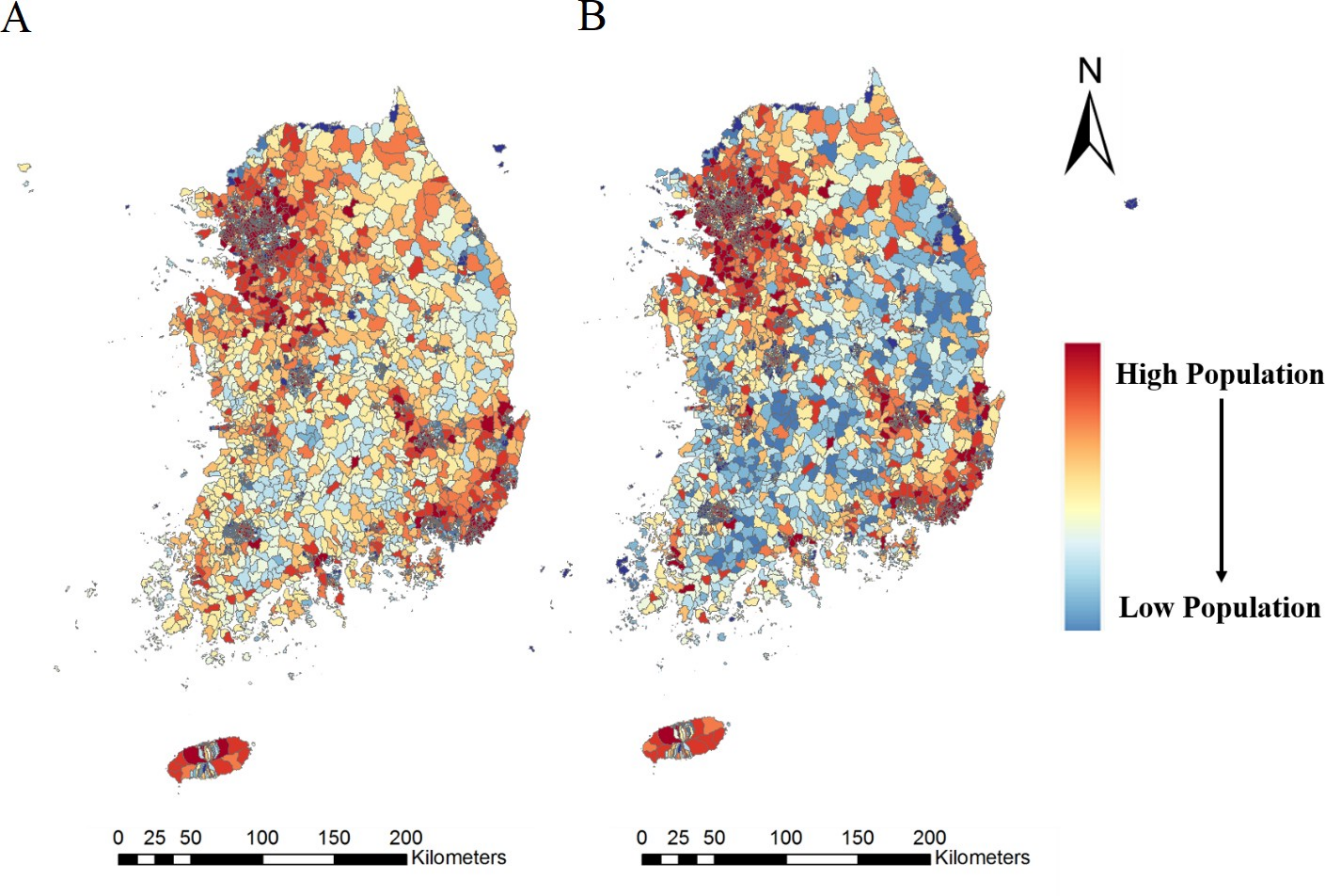
Map of population. **(**A) Census-based population. (B) Mobile-based population.

**Fig 5:**
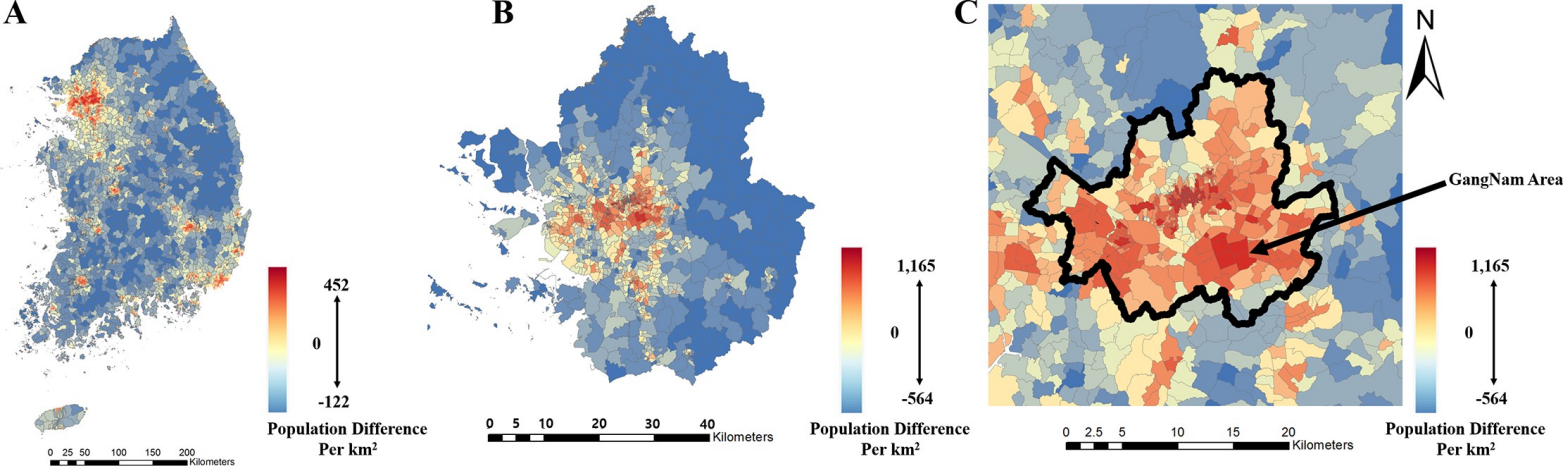
Differential map between census-based and mobile-based population count (mobile-based minus census-based). (A) South Korea. (B) The Seoul metropolitan area. (C) Seoul in black boundary.

Fig 4 shows maps of the entire South Korean population in the forms of a census-based population map and a mobile-based population map. Seoul city shows a high population in both the census and population data, while rural areas show some differences between the two types of population data. The 50m by 50m mobile-based population data were aggregated into ‘Dong’ district spatial unit for comparison. Fig 5 was derived by calculating the difference between the two population data sets. The areas with negative population differences between the census-based and mobile-based populations show that there is more census-based population than mobile-based population, depicting those areas as having low levels of activity. It is because that census-based population data describe where people live, in terms of demographics acquired by the government, thus showing where each person would reside after active hours. Whereas mobile-based population data illustrate the location of active personnel, which in turn characterize location in which a person would perform their daily lives. Contrastingly, the areas with positive population differences depict a high level of activity, which in turn implies ‘more people are working and doing activities in the area than residing at home.’ Fig 5 also includes differential maps of the Seoul metropolitan area and of the Seoul area. The arrow in Fig 5C indicates the Gangnam area, which has a very high mobile-based population compared to the census-based population, since it consists mostly of business sectors and cultural spaces.

### Network-based service areas

**Fig 6:**
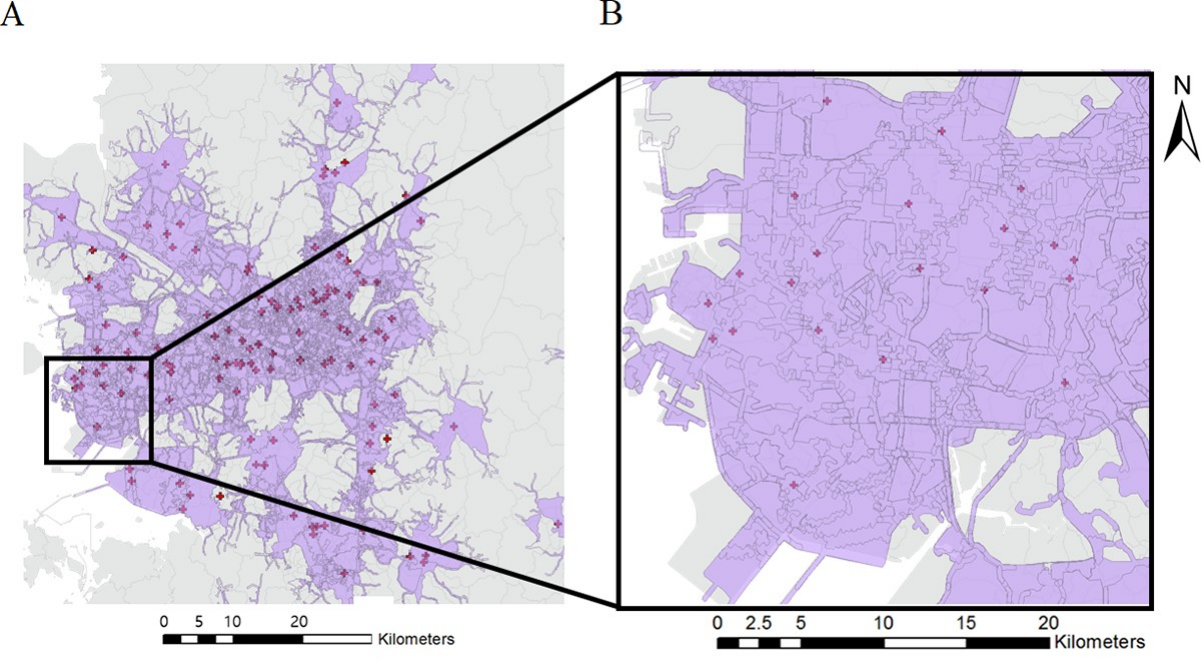
Network-based service area with 10-min. threshold. (A) Around Seoul, South Korea. (B) Incheon area.

The results from the network-based service area analysis are shown in Fig 6. The figure depicts areas around Seoul and Incheon within the 10-minute threshold for emergency services. As seen in the figure, the service area analysis made use of network data to calculate the ‘reachable’ area within the given threshold. Since the method is ‘network based’, some areas could be excluded due to lack of network roads, even though they are within the reachable distance. In this section, only the service area results for the emergency care locations are shown, since the ‘Dong’-based service area results cover the entire map.

### Accessibility analysis result

**Fig 7:**
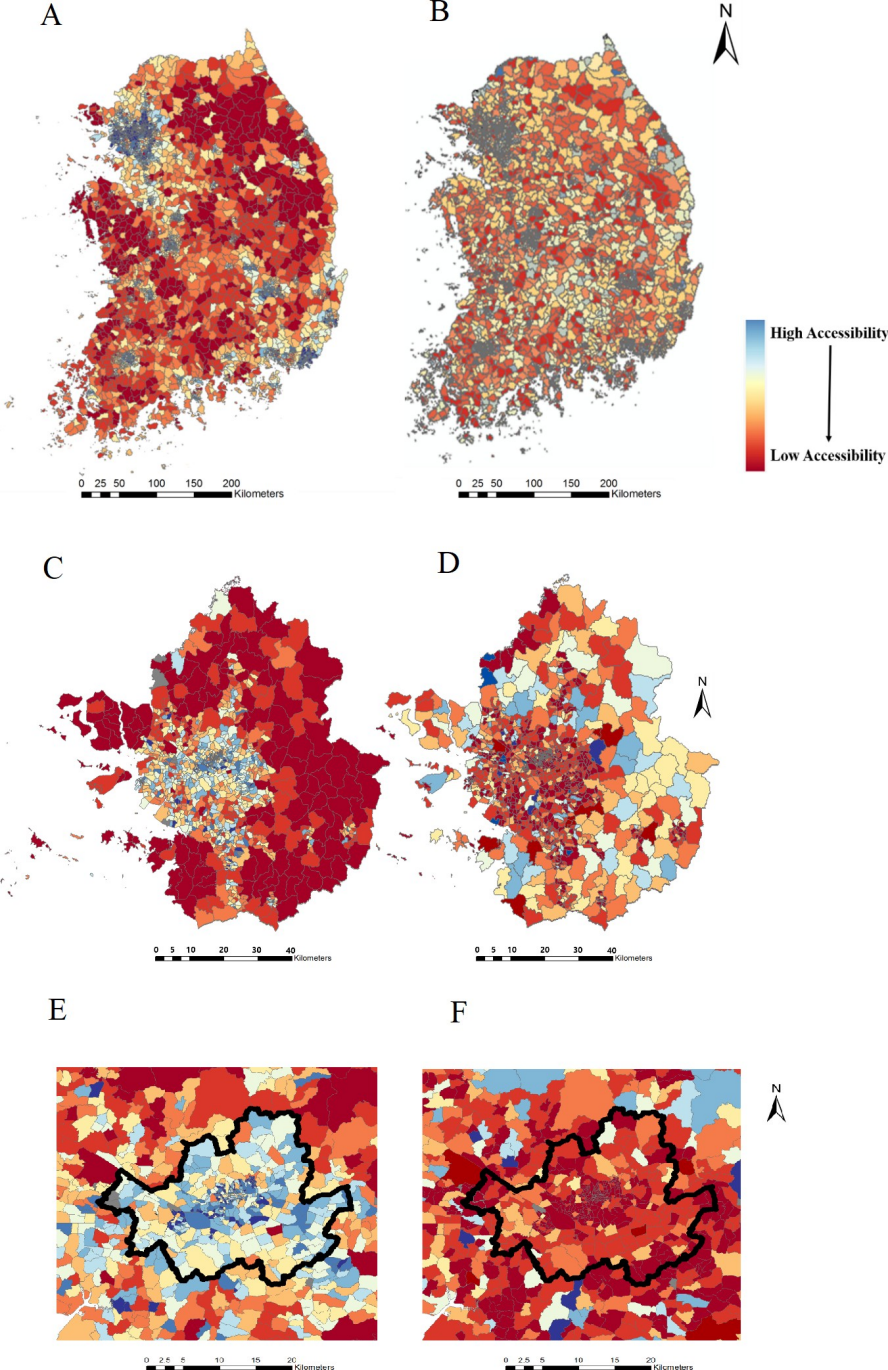
Map of accessibility to emergency room with 10-min. threshold. (A) South Korea by census-based population. (B) South Korea by mobile-based population. (C) The Seoul metropolitan area by census-based population. (D) The Seoul metropolitan area by mobile-based population. (E) Seoul area by census-based population. (F) Seoul area by mobile-based population.

**Fig 8:**
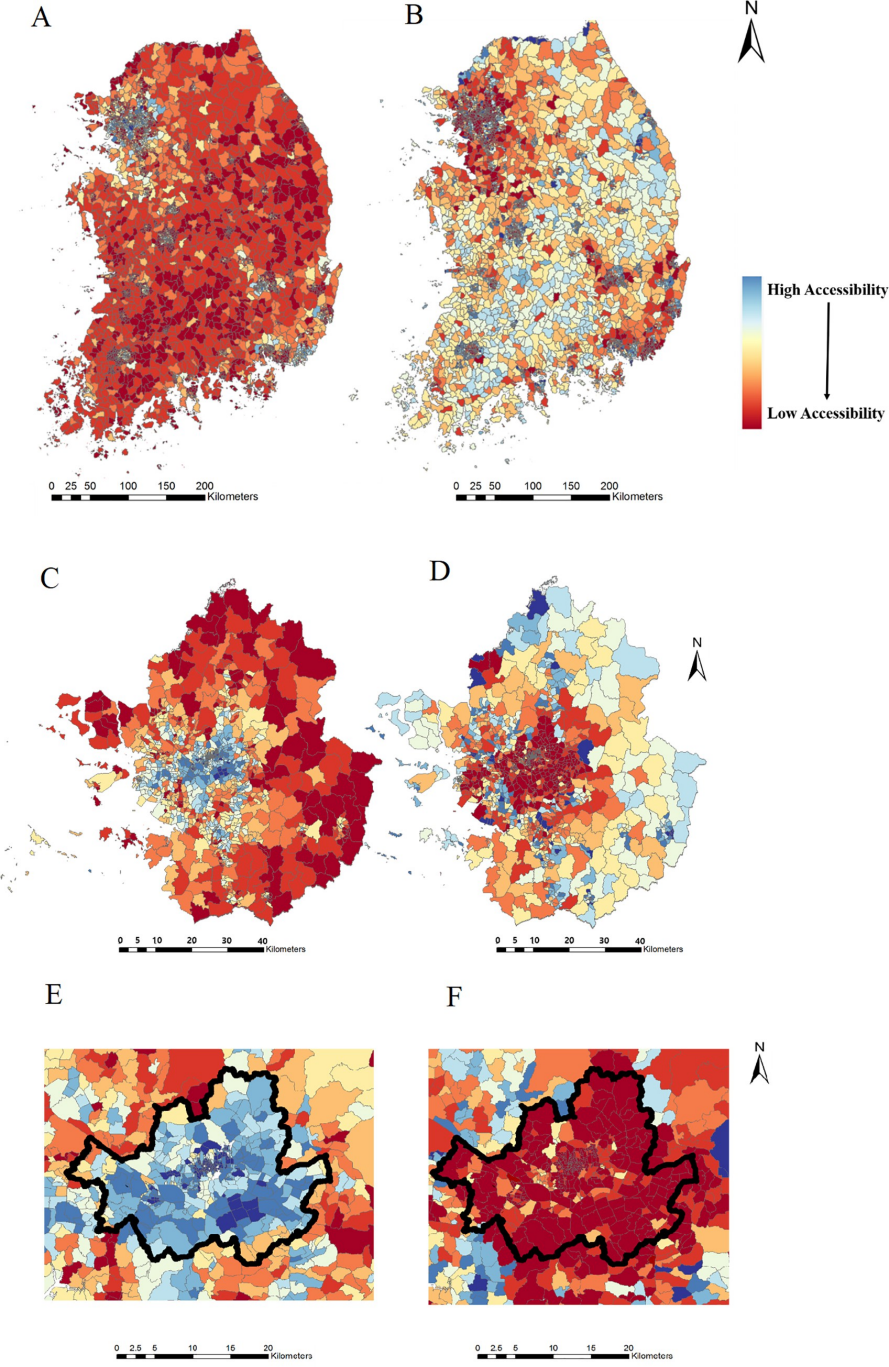
Map of accessibility to emergency room with 15-min. threshold. (A) South Korea by census-based population. (B) South Korea by mobile-based population. (C) The Seoul metropolitan area by census-based population. (D) The Seoul metropolitan area by mobile-based population n. (E) Seoul area by census-based population. (F) Seoul area by mobile-based population.

The results of the 2SFCA are represented for South Korea, the Seoul metropolitan area, and the Seoul area, respectively in Figs 7 and 8. As noted earlier, the distance (service area) threshold was set to 10 and 15 minutes in consideration of the ‘platinum 10 minutes’ for emergency patient transport to emergency room facilities. Fig 7 utilized 10 minutes as the threshold and Fig 8 utilized 15 minutes as the threshold. For an unbiased comparison between the census-based and the mobile-based analyses, percentile of accessibility value was used for map representation, instead of actual accessibility value.

## Discussion

All the analysis results showed clear distinctions between the two different data sets. The empirical results gave the answers to our hypotheses.

As to whether there is a difference in the geographical distribution of accessibility when using two population data sets, our results showed that the accessibility of emergency rooms measured by census-based population data was different from that by mobile-based population data in South Korea. The results for the census-based population showed high accessibility in urban areas and very low accessibility in rural areas, whereas the results for the mobile-based population showed low accessibility in urban areas and high accessibility in rural areas.

Fig 7 depicts the accessibility score for the whole of South Korea. As seen in Fig 7A and 7B, Gangwon-do, at the top right region of South Korea, showed very low accessibility for emergency medical care by census-based population data while the accessibility score for the Gangwon-do area is comparatively higher by mobile-based population data. The difference in the results was due to the differing characteristics of the census-based and mobile-based populations. The census-based population can show only the number of people who are registered in the district and cannot reflect actual details that can be captured by the mobile-based population. As seen in Fig 4, the population in the rural area of Gangwon-do shows a comparatively high census-based population (Fig 4A) and a comparatively low mobile-based population (Fig 4B). The difference between the two types of populations, as depicted in Fig 5, can be used to explain the difference in accessibility scores between census-based and mobile-based populations in Figs 7 and 8. Due to the higher census-based population and lower mobile-based population, in South Korea, the census-based accessibility maps in Figs 7A, 7B and 8A, and 8B show low accessibility index scores for the Gangwon-do area; however, the low mobile-based population causes the accessibility index to increase. In a previous study that attempted to statistically represent emergency room usage in given areas [49], the emergency rooms in Gangwon-do showed very low usage. In that study, the usage rate was acquired by calculating the number of patients using emergency care, thus implying that the Gangwon-do area, even with the lowest number of emergency rooms, has sufficient emergency care services. This coincides with the accessibility index results derived from the mobile-based population and shows that the mobile-based population reflects reality more accurately.

Another phenomenon can be seen in the Seoul metropolitan area in Figs 7C, 7D, 8C and 8D. The two maps also show a very stark difference in accessibility scores. The Seoul area showed very high accessibility when the census-based population was used, but rather low accessibility when the mobile-based population data were used, whereas the outskirts of Seoul showed very low accessibility with the census-based population and high accessibility with the mobile-based population. Fig 5B, which shows the difference between the mobile- and census-based populations, can be referred to in order to explain the difference between the two maps of the Seoul metropolitan areas in Figs 7 and 8. The Seoul area contains a higher mobile-based population than census-based population, which coincides with the fact that when using the mobile-based population, the demand for emergency rooms would rise, causing the accessibility index to decrease. The outskirts of Seoul, in contrast, are mainly ‘bedroom communities’ and generally contain a lower mobile-based than census-based population, which would cause the opposite effect in the accessibility score when compared with the Seoul area.

The difference between Seoul and the outskirts can be seen more closely in Figs 7E, 7F, 8E and 8F, since the Seoul areas contain the highest density of hospitals as well as highest mobile-based populations. This causes the census-based population result to show a very high accessibility index, due to the large number of hospitals; however, when the mobile-based population is considered, the result drastically changes, due to the high density and high mobile-based population in the Seoul area. The urban- and rural-area results in the present study coincided in their accessibility results derived by using mobile-based population data, thus proving that usage of mobile-based population data for accessibility analysis enables more accurate interpretation of real-world situations.

On the other hand, for the hypothesis that there is a difference between setting driving of the vehicle to 10 and 15 minutes, the results indicated that the geographical inequality in the accessibility to emergency rooms by the 15-minute threshold for service areas was more significant than in the case of the 10-minute threshold.

Fig 7 shows the results of the 10-minute threshold for service areas within all of South Korea, the Seoul metropolitan area, and Seoul, respectively, and Fig 8 shows that of the 15-minute threshold. When the 15-minute threshold for service area was used to evaluate the accessibility index (Fig 8), the area coverage for supply and demand was increased compared with the 10-minute case (Fig 7), thus covering more areas with larger census-based and mobile-based populations, as well as more supplies to be shared.

As for the results for the census-based population, increasing the service threshold from 10 to 15 minutes caused rural areas to show lower accessibility indices all around, and improved the accessibility index for the Seoul area. On the other hand, with respect to the mobile-based population, increasing the threshold presented the opposite results: rural areas had higher accessibility indices, and the Seoul area had a lower accessibility index. The decreased accessibility index for the increased time threshold with a mobile-based population for Seoul could have been due to the high mobile-based population density in the Seoul area, the capital city of Korea. The mobile-based population of the Seoul area has been calculated to be around 11.51 million out of the 45.21 million mobile-based population of the whole of South Korea, which is 25.4%. Since the 2SFCA algorithm in Eq 2 utilizes census and mobile-based populations within the threshold (10 or 15 minutes) as the demand, the gradient of demand *D*_*k*_, when the service threshold is increased from 10 to 15 minutes, is much higher for the Seoul area than for rural areas. The Seoul area, with its much higher mobile-based population density, would see the value of *D*_*k*_ increasing drastically when the 15-minute threshold is used. This would cause the accessibility score to be far lower, as shown in Fig 7 compared with Fig 8. On the other hand, in rural areas, the gradient of *D*_*k*_ would be smaller than in the Seoul area, due to the less dense populations, causing the accessibility score to fluctuate only in small values.

The different thresholds of 10 and 15 minutes affect both the census-based population and the mobile-based population results in opposite ways. For the mobile-based population, use of the 15-minute threshold increases the accessibility index in rural areas significantly, whereas it significantly decreases the accessibility index in the Seoul area. As for the census-based population results, the accessibility index in rural areas was decreased, and that in the Seoul area was increased.

In the entire country of South Korea, the accessibility index difference between the census-based and mobile-based populations with the 15-minute threshold is larger than that with 10-minute threshold as shown in Figs 7A, 7B, 8A and 8B. When the accessibility index for the Seoul metropolitan area was calculated with the 15-minute threshold, the difference between the census-based and mobile-based population results in Fig 7C and 7D was amplified in Fig 8C and 8D, showing high accessibility for the census-based population and very low accessibility for the mobile-based population. This phenomenon can also be seen in the Seoul area (Figs 7E, 7F, 8E and 8F). These results would suggest that the 15-minute threshold reflects a more urgent need to supplement the emergency rooms; however, further study seeking to describe the behavior of emergency patients would require an optimal time threshold.

As mentioned in section 1, most of the previous studies have utilized census-based populations as the demand input for 2SFCA. However, the results of the present study clearly show that usage only of a census-based population could lead to misleading results for emergency medical care accessibility. Therefore, using an additional, mobile-based population in accessibility analysis can be crucial for more rational analysis, since it can create more ‘real-world’ results. The newly acquired results could help governments and the private sector to make better decisions and fairer policies in support of hospitals, or even to construct new hospitals for better accessibility for citizens. In effect, this study can help to derive solutions to problems of social injustice in health care.

## Conclusions

This study aimed to provide new insights for medical care accessibility analysis by utilizing mobile-based population data as the demand side of emergency rooms. The 2SFCA algorithm was implemented for service areas derived using network-based service area analysis, and the two results, elaborated from using census-based and mobile-based population data sets, respectively, were compared.

The results derived from the census-based population data showed major medical care accessibility inadequacy in the Gangwon-do region. This area consists of mountains and farming areas, due to which fact, insufficiency might be overestimated, since fewer medical facilities are present there. The results obtained from the mobile-based population data showed less insufficiency, since those data reflected the actual situation (i.e., fewer people being situated within the rural Gangwon-do area). This difference affected by use of the mobile-based population could be clearly seen.

Another major difference between the two results could be seen in the urban areas including Seoul. As shown in Figs 7E, 7F, 8E and 8F, wherein the Seoul area is highlighted with black boundaries, when the census-based population data were used, the Seoul area showed high accessibility of medical care. On the other hand, when the mobile-based population data were implemented, the accessibility decreased drastically. This phenomenon could have been due to the characteristics of the Seoul area. Being the capital of South Korea, Seoul contains a very large number of businesses. Many of the people living on the outskirts of Seoul commute to Seoul to work every day, leading to a higher mobile-based population inside Seoul when compared with the census-based population of Seoul. Reinforcing this finding, the outskirts of Seoul showed low accessibility with the census-based population data and rather higher accessibility with the mobile-based population data. When the 15-minute service threshold was used, the results were intensified, showing a clearer difference between the census-based and mobile-based populations. As for the results from the census-based population data, the rural area showed a bigger deficit for emergency rooms, and the Seoul area showed a larger increase of accessibility. For the mobile-based population data by contrast, the rural area showed a larger increase of accessibility, and the Seoul area showed a bigger deficit of emergency rooms.

By using mobile-based population data, along with census-based population data, and comparing the respective results derived from them, new insights for urban and rural areas could be constructed. The findings of this study could be utilized in order to resolve emergency medical care inequality between regions; certainly, they could aid decision making and policy formulation.

On the other hand, research will also be needed to improve the accuracy and reliability of mobile-based population data. Considering the high usage rate of mobile phone in Korean adults, those who do not own mobile phones would be very young or very old people. A method for data correction for a specific age group without detracting from the reliability of the whole data should be investigated. It would be also necessary to investigate whether there is a relationship between a particular service provider and the region or income. Moreover, it would be possible to examine whether the errors would occur by the difference of distribution of base stations or the mobile phone usage patterns between in urban and in rural areas.

Further studies will attempt to compensate for the current study’s limitations. Road network data provided by ITS does not contain unpaved roads in rural areas. These ‘unregistered’ road network data are usually managed by local government sectors. Thus, by acquiring data from local government sectors, more thorough analysis can be conducted. Also, when calculating service areas by 2SFCA, the time threshold was set as 10 and 15 minutes and the speed of moving cars was 40km/h. This limitation could be further removed by considering real-time traffic conditions and the behaviors of emergency patients. This study might also contain MAUPs (modifiable areal unit problems), since the accessibility score was aggregated according to ‘Dong’ administrative districts. The MAUP problem [50] can be further analyzed by implementing various types of area-aggregation techniques and various administrative district units and will be studied in the near future.

Also, instead of overall monthly average population counts, other counts from different time segmentations, such as weekdays, weekends, daytime, and nighttime, could be used to show more detailed and realistic patterns of movement under different circumstances, which will be further analyzed in an upcoming study. In addition, various accessibility measures, such as Enhances 2-Step Floating Catchment Analysis (E2SFCA) and 3-Step Floating Catchment Analysis (3SFCA), could be implemented for a more thorough results comparison instead of the 2SFCA. Accessibility within the catchment area could be differentiated since E2SFCA uses multiple distance decay weights rather than binary weights as in 2SFCA. Also, 3SFCA was introduced to minimize the demand overestimating problem of E2SFCA. By applying these methods, more realistic analysis would be expected. Lastly ‘inverted-2SFCA’ method could be further utilized to see the actual usage of each facility [51]. The index measures the crowdedness for facilities by checking scarcity of resource or intensity of competition.

## Abbreviations

2SFCA: 2-Step Floating Catchment Analysis
EMS: Emergency Medical Service
